# Supplementary material for: Identification of a novel deFADding activity in human, yeast and bacterial 5′ to 3′ exoribonucleases
Source: Nucleic Acids Res. 2022 Jul 29;50(15):8807–17. doi: 10.1093/nar/gkac617 (PMC9410882; doi:10.1093/nar/gkac617)
Supplement: gkac617_Supplemental_Files [file gkac617_supplemental_files.zip › SupplementaryDATA_rev.pdf]

Identification of a novel deFADding activity in 5′ to 3′ exoribonucleases.

Sunny Sharma†[1], Jun Yang†[1], Selom K. Doamekpor[2], Ewa-Grudizen Nogalska[1], Liang Tong[2], and Megerditch Kiledjian[1*]

[1] Department of Cell Biology and Neurosciences, Rutgers, University, Piscataway, New Jersey, 08854, USA

[2] Department Biological Sciences, Columbia University, New York, NY 10027, USA

[*]To whom correspondence should be addressed. Tel: +1 848 445 0796; Email: kiledjian@biology.rutgers.edu [†]Joint First Authors.

Supplementary Figures

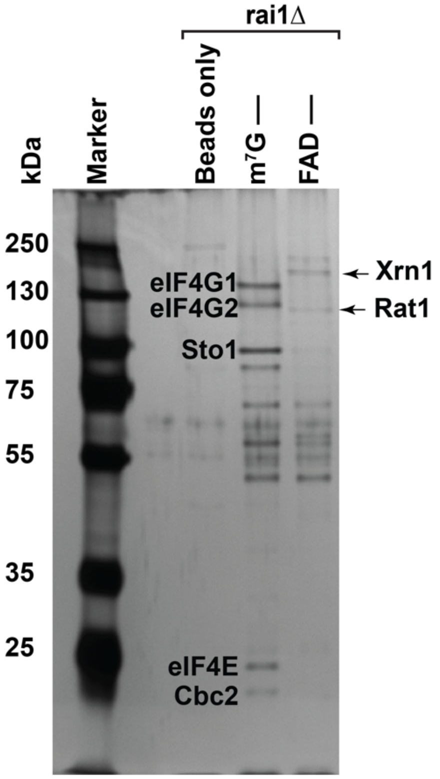

Supplementary Figure S1. Characterization of proteins bound to the FAD cap in the absence of Rai1. Protein derived from a strain lacking Rai1 (*rai1*Δ) were captured by FcRAP and eluates detected by Silver Staining following resolution on 10% SDS-PAGE gel. Affinity purification with 5′ m^7G capped RNA (m^7G–) or FAD-capped RNA (FAD–) are shown. Streptavidin beads without RNA baits (beads only) was used a control.

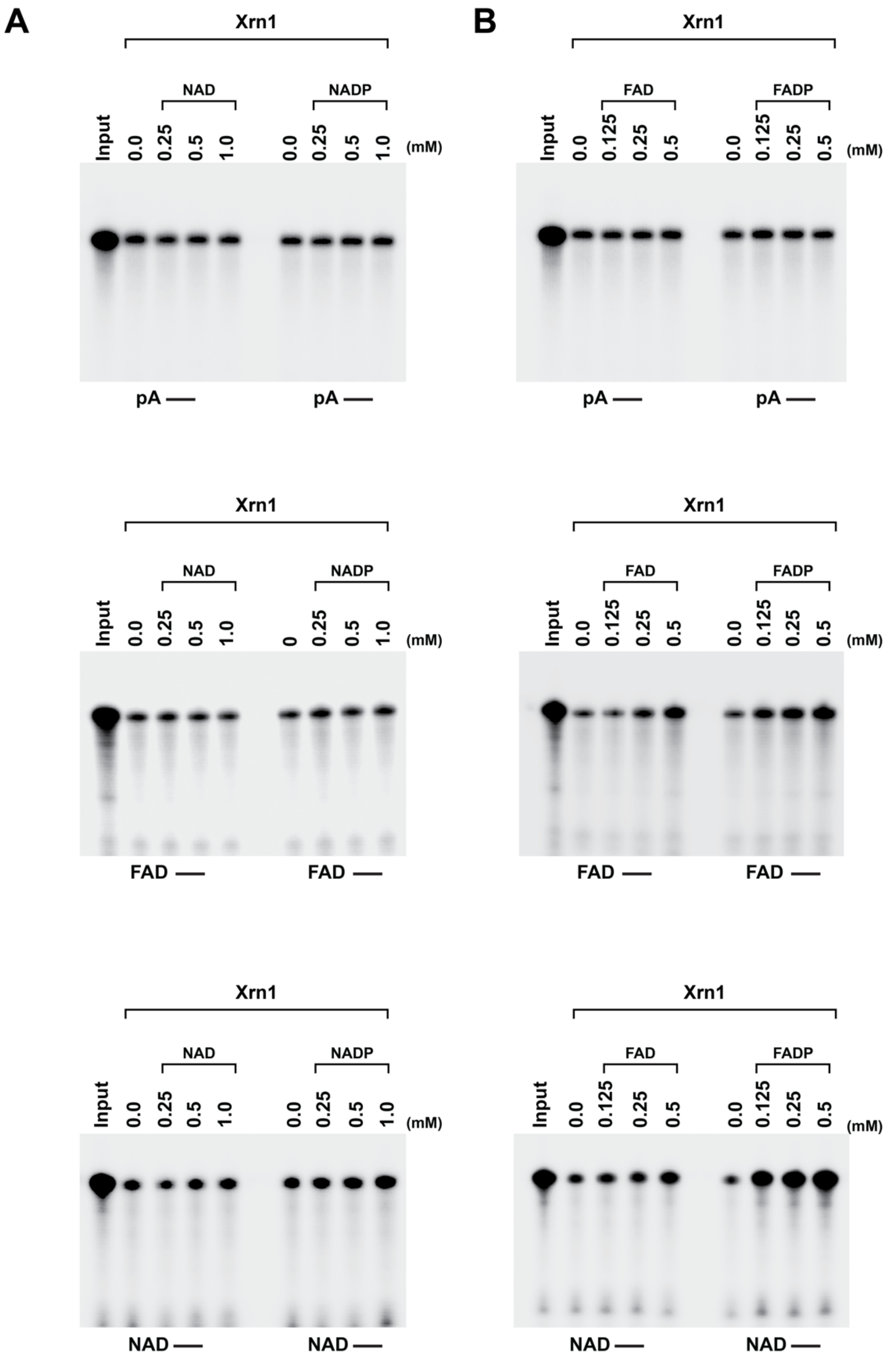

Supplementary Figure S2. Xrn1 possess higher deFADding activity compared to its deNADding activity. To define the differential preference of FAD- over NAD-capped RNA by Xrn1, its degradation activity was deciphered in the presence of competitors (free NAD or NAD3′p and free FAD and FAD3’p, which would mimic the 5′ end of an NAD-capped or FAD-capped RNA, respectively. 100nM recombinant Xrn1 from *K. lactis* was incubated with uniformly ^{32}P-labeled 5′- monophosphate (pA–), NAD-capped or FAD-capped RNA in the

presence or different amount of free NAD and NAD3'p **(A)**, FAD and FAD3'p **(B).** The products were resolved on 15% 7M urea PAGE gels

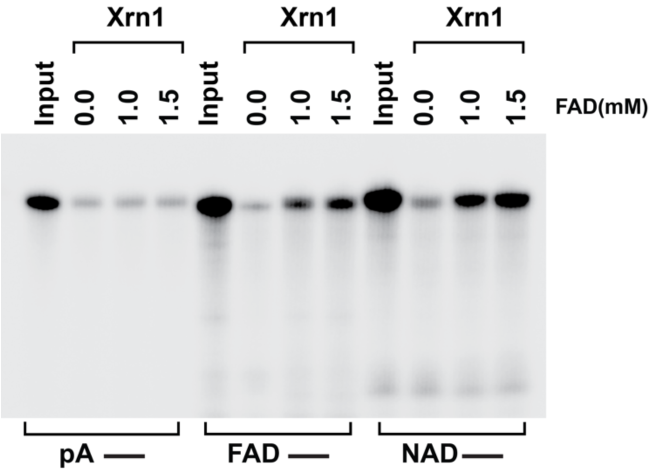

Supplementary Figure S3. Higher concentration of free FAD inhibits deNADding and deFADding activity of Xrn1. 100nM recombinant Xrn1 from *K. lactis* was incubated with uniformly ^{32}P-labeled 5′- monophosphate (pA–), NAD-capped or FAD-capped RNA in the presence or different amount of free FAD**.** The products were resolved on 15% 7M urea PAGE gels.

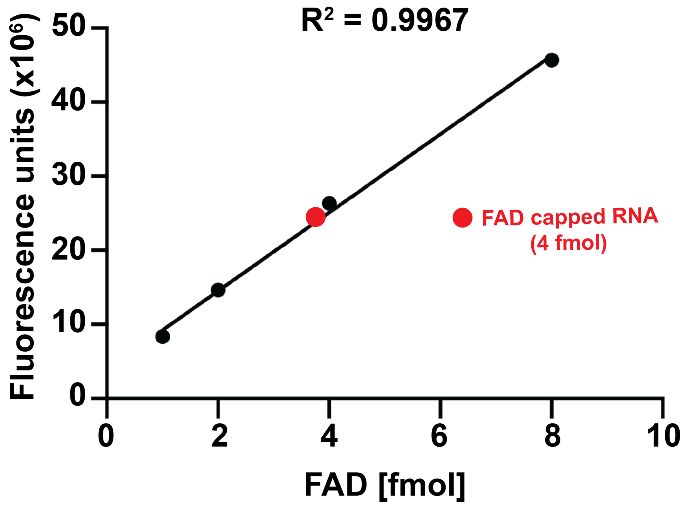

Supplementary Figure S4. Comparison of FAD released from FAD-capped RNA to that of free FAD. The standard curve obtained after plotting the fluorescent units against the amount of free FAD using FAD-capQ assay. Fluorescent units obtained after digesting 4 fmol of in vitro transcribed FAD capped RNA is also plotted on the curve and is denoted as red circle.

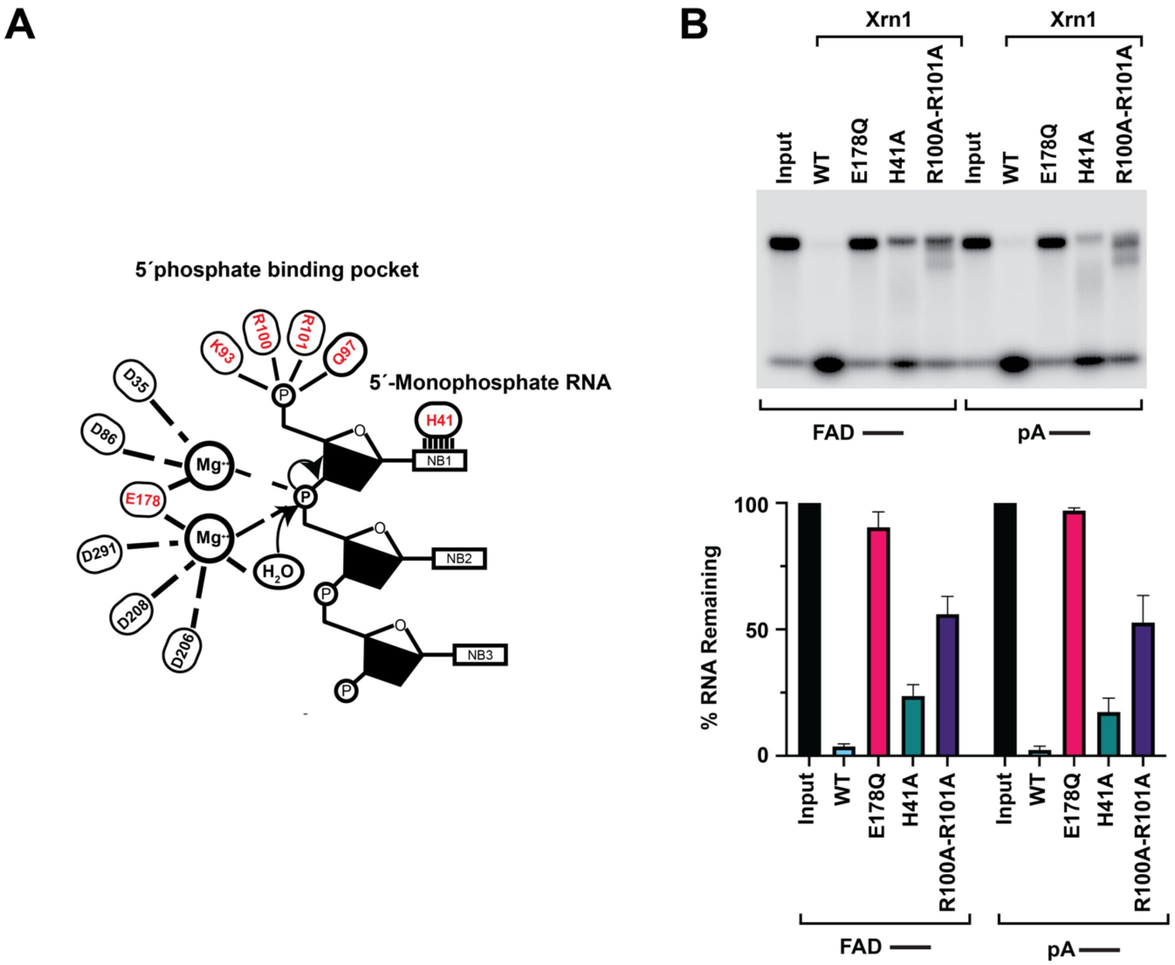

Supplementary Figure S5. The xrn1-H41A point mutant is dispensable for FAD capped hydrolysis (A) Model for monophosphate RNA hydrolysis (Jinek et al 2012). As shown recently (Sharma et al 2022), H41A abolishes the deNADding activity of Xrn1without affecting its 5' monophosphate RNA hydrolysis. Surprisingly, as for monophosphate RNA, FAD capped RNA also remained unaffected upon mutating H41A.**(B)** 100nM recombinant WT or different point mutated Xrn1 from *K. lactis* was incubated with uniformly ^{32}P-labeled 5′-monophosphate or FAD-capped RNA. The products were resolved on 15% 7M urea PAGE gels. Quantitation of RNA remaining is plotted from three independent experiments with ± SD denoted by error bars

Supplementary Table S2. Oligonucleotides used in the present study

NAME	SEQUENCE 5'->3'	PURPOSE
ϕ2.5-NAD/FAD-40	CAGT**AATACGACTCACTATT**AGTTGGTGGTTGTTGTGTGTTTGTGGTTGGTTTGTTTGGC	In vitro transcription (T7 promoter sequence is highlighted)
PET-BACYV-A1	TAAGAAGGAGATATACCATGAGCGACACGAATTATGCAGTGAT	For cloning YciV ORF in pET plamsids
PET-BACYV-A2	GTGGTGGTGGTGGTGCTCGAGTAATTCCCTCTCTGTGGTGTTCTGCG	For cloning YciV ORF in pET plamsids
YCIV-D20A-A1	CAGCTTCCGcTGGCTGCCTGACGCCAGAAG	For site directed mutagenesis (pET-rnase AM-D20A)
YCIV-D20A-A2	CAGGCAGCCAgCGGAAGCTGTGGTATGGC	For site directed mutagenesis (pET-rnase AM-D20A)
YCIV-D255AH257A-A1	GGATCTGcTTTTgcTCAGCCATGCCCGTGGATCG	For site directed mutagenesis (pET-rnase AM- D255AH257A)
YCIV-D255AH257A-A2	GGCTGAgcAAAAgCAGATCCTTGTGATGCCCATAAATG	For site directed mutagenesis (pET-rnase AM- D255AH257A)
YCIV-E72A-A1	CCCGGCGTGGcAATTTCCACGGTCTGGG	For site directed mutagenesis (pET-rnase AM- E72A)
YCIV-E72A-A2	CGTGGAAATTgCCACGCCGGGAATAAGATTC	For site directed mutagenesis (pET-rnase AM- E72A)

Supplementary Table S3. Reagents used in the present study

REAGENT or RESOURCE	SOURCE	IDENTIFIER
**Yeast/E.coli Strains and Human Cell lines**		
BY4741 (MATa; his3Δ1; leu2Δ0; met15Δ0; ura3Δ0)	Dharmacon	YSC1048
xrn1Δ (BY4741; MATa; his3Δ1; leu2Δ0; met15Δ0; ura3Δ0; Xrn1: KanMX	This study	NA
E. coli K-12 BW25113	Horizon Discovery	OEC5042
yciv/rnase AM-KO	Horizon Discovery	OEC4987-200826696
HEK293T	Gilbertson et al (2018) (1)	NA
XRN1-KO	Gilbertson et al (2018) (1)	NA
**Plasmids**		
pET-KlXrn1 (A derivative pET26a(+) plasmid carrying KlXrn1(1,1245)-6xHis fusion protein)	Chang et al(2011) (2)	NA
pET-klxrn1-H41A (A derivative pET-KlXrn1 plasmid carrying mutant klxrn1-H41A-6xHis fusion protein)	Sharma et al (2022) (3)	
pET-RNAse AM (A derivative pET26a(+) plasmid carrying YciV(ORF)-6xHis fusion protein	This study	NA
pET-rnase AM-D20A (A derivative pET26a(+) plasmid carrying mut carrying mutant yciV-D20A -6xHis fusion protein	This study	NA
pET- rnase AM-E72A (A derivative pET26a(+) plasmid carrying mutant yciV-E72A -6xHis fusion protein	This study	NA
pET- rnase AM-D255A-H257A (A derivative pET26a(+) plasmid carrying mutant yciV- D255A-H257A -6xHis fusion protein		
pET-klxrn1-K93A (A derivative pET-KlXrn1 plasmid carrying mutant klxrn1-K93A-6xHis fusion protein)	Sharma et al (2022) (3)	NA
pET-klxrn1-Q97A (A derivative pET-KlXrn1 plasmid carrying mutant klxrn1-K97A-6xHis fusion protein)	Sharma et al (2022) (3)	NA
pET-HsXrn1 (A derivative pET26a(+) plasmid carrying HsXrn1(1,1189)-6xHis fusion protein)	Sharma et al (2022) (3)	NA
pET-HsXrn1-E178Q (A derivative pET26a(+) plasmid carrying mutant HsXrn1-E178Q(1,1189)-6xHis fusion protein)	Sharma et al (2022) (3)	NA
pET-klxrn1-R100/101A (A derivative pET-KlXrn1 plasmid carrying mutant klxrn1-R100A, R101A-6xHis fusion protein)	Sharma et al (2022) (3)	NA

References

1. Gilbertson, S., Federspiel, J.D., Hartenian, E., Cristea, I.M. and Glaunsinger, B. (2018) Changes in mRNA abundance drive shuttling of RNA binding proteins, linking cytoplasmic RNA degradation to transcription. *Elife*, **7**.
2. Chang, J.H., Xiang, S., Xiang, K., Manley, J.L. and Tong, L. (2011) Structural and biochemical studies of the 5'-->3' exoribonuclease Xrn1. *Nat Struct Mol Biol*, **18**, 270-276.
3. Sharma, S., Yang, J., Grudzien-Nogalska, E., Shivas, J., Kwan, K.Y. and Kiledjian, M. (2022) Xrn1 is a deNADding enzyme modulating mitochondrial NAD-capped RNA. *Nat Commun*, **13**, 889.
